# Complete Genome Sequences of T4-Like Bacteriophages RB3, RB5, RB6, RB7, RB9, RB10, RB27, RB33, RB55, RB59, and RB68

**DOI:** 10.1128/genomeA.01122-14

**Published:** 2015-01-02

**Authors:** Stephanie J. Yaung, Kevin M. Esvelt, George M. Church

**Affiliations:** a Wyss Institute for Biologically Inspired Engineering, Harvard Medical School, Boston, Massachusetts, USA; b Department of Genetics, Harvard Medical School, Boston, Massachusetts, USA; c Program in Medical Engineering & Medical Physics, Harvard-MIT Division of Health Sciences and Technology, Massachusetts Institute of Technology, Cambridge, Massachusetts, USA

## Abstract

T4-like bacteriophages have been explored for phage therapy and are model organisms for phage genomics and evolution. Here, we describe the sequencing of 11 T4-like phages. We found a high nucleotide similarity among the T4, RB55, and RB59; RB32 and RB33; and RB3, RB5, RB6, RB7, RB9, and RB10 phages.

## GENOME ANNOUNCEMENT

The complete sequences of T4-like myoviruses would enhance studies of phage evolution and genomics, as well as biotechnology applications involving phage cocktails. In this study, we sequenced the genomes of the RB3, RB5, RB6, RB7, RB9, RB10, RB27, RB33, RB55, RB59, and RB68 phages. The RB phages were originally isolated by Rosina Berry in 1964 from six sewage treatment plants in Long Island, NY, for studies on speciation among T-even phages (1).

We prepared phage lysates as previously described (2) from host Escherichia coli B (CGSC 5365), extracted DNA with the Phage DNA isolation kit (bioWORLD, Dublin, OH), and sequenced the samples as paired-end 250-bp reads on the MiSeq instrument (Illumina, San Diego, CA). A total of 789,300 (for RB6) to 3,932,449 (for RB7) paired reads were generated per sample. On average, 82.8% of the pairs survived quality control and trimming with Trimmomatic (3). The insert sizes were ~330 bp; the median coverage of sequenced phages was 2,966×, ranging from 259× (for RB55) to 6,985× (for RB7). We performed *de novo* assembly using Velvet version 1.2.08 (4), with *k*-mer lengths of K51, K57, and K63, and we were able to obtain a single ~168-kbp contig from at least one of the assemblies. We used Geneious version 7.1.7 for postassembly processing and filled any assembly gaps by iterative mapping of reads to the scaffold.

The circularly permuted linear double-stranded DNA genomes of the 11 RB phages have lengths of ~168 kbp. Approximately 270 open reading frames (ORFs) per phage were predicted with Glimmer 3 (5). The annotations were transferred from the published genomes of T4 and T4-like phages with at least 98% similarity. The remaining ORFs were annotated by lowering the similarity cutoff to 70% or performing BLAST searches (6). Eight to 10 tRNAs were predicted in each genome by tRNAscan-SE 1.21 (http://lowelab.ucsc.edu/tRNAscan-SE/ [7]). According to the convention in T4-like phages, we oriented completed genomes to start with rIIA.

The sequenced phages have a similar genome organization and nucleotide identity. Using progressiveMauve alignment (8), we found that RB7, RB27, RB33, and RB68 are 73 to 86% similar to one another and are ~75% identical to T4. Furthermore, RB33 shares 99.93% similarity with RB32. RB55 and RB59 are 99.8% similar to T4 and are 99.96% identical to each other. We noted a high nucleotide similarity (99.99%) among RB3, RB5, RB6, RB7, RB9, and RB10. RB5 differs from RB6 by four bases (one nonsynonymous, one synonymous, and two intergenic); the nonsynonymous difference occurs in the baseplate wedge subunit and tail pin, gene product 11 (gp11). RB7 and RB9 differ by three nucleotides (two nonsynonymous and one intergenic); the two nonsynonymous bases are in the baseplate hub subunit tail length determinator (gp29) and hypothetical protein NrdC.4. The extent to which these differences affect host range is unclear given the limited data on the total number but not the exact profile of susceptible E. coli strains within the Escherichia coli Collection of Reference (ECOR) collection for each phage (9). The relationships between genome and host range variation might provide insights into mechanisms of host specificity.

### Nucleotide sequence accession numbers.

The genome sequences have been deposited in GenBank, and the accession numbers are listed in Table 1.

**TABLE 1:** Genome features of the sequenced strains

Enterobacteria phage	Accession no.	Genome size (bp)	Coverage (×)	No. of CDSs^*a*^	No. of tRNAs
RB3	KM606994	168,402	2,831	273	10
RB5	KM606995	168,394	3,449	271	10
RB6	KM606996	168,394	1,474	271	10
RB7	KM606997	168,395	6,985	272	10
RB9	KM606998	168,395	2,826	272	10
RB10	KM606999	168,401	2,798	272	10
RB27	KM607000	165,179	2,966	271	10
RB33	KM607001	166,007	3,355	274	8
RB55	KM607002	168,896	259	272	8
RB59	KM607003	168,966	3,158	276	8
RB68	KM607004	168,401	3,187	276	9

^a^ CDSs, coding sequences.
